# Clinical Presentations, Diagnosis, and Management for Pediatric Antral Web—A 20-Year Experience of a Referral Center

**DOI:** 10.3389/fped.2021.753076

**Published:** 2021-12-14

**Authors:** Pai-Jui Yeh, Hsun-Chin Chao, Chien-Chang Chen, Jin-Yao Lai, Ming-Wei Lai

**Affiliations:** ^1^Division of Pediatric Gastroenterology, Department of Pediatrics, Chang Gung Memorial Hospital, Linkou Branch and Chang Gung University College of Medicine, Taoyuan, Taiwan; ^2^Department of Pediatric Surgery, Chang Gung Memorial Hospital, Linkou Branch and Chang Gung University College of Medicine, Taoyuan, Taiwan; ^3^Liver Research Center, Chang Gung Memorial Hospital, Linkou Branch, Linkou, Taiwan

**Keywords:** antral web, children, surgery, intervention, gastric outlet obstruction

## Abstract

**Background:** Antral web is a rare cause of gastric outlet obstruction in children. The presentation is diverse, depending on the degree of obstruction. Unfortunately, the guidance of management is still lacking.

**Methods:** This study retrospectively evaluated the presentations, management, and outcomes of the pediatric antral web based on a 20-year experience in a referral center.

**Results:** A total of 23 cases were included. The median age of diagnosis was 10 months (interquartile range, IQR, 0.8–23 months). Main presentations comprised vomiting (83%) and upper gastrointestinal (UGI) bleeding (48%). Concurrent gastric ulcers were common (68%). A total of 13 cases (57%) underwent interventional treatment. The median duration from diagnosis to intervention (DtI) was 10 days, but five with longer DtI, ranged from 30 to 755 days. Among the 15 cases with concurrent gastric ulcers, 10 patients received intervention, immediately in six but delayed in four. Surgical treatments (*N* = 12) achieved a cure in 11, with one rescued by endoscopic treatment.

**Conclusions:** Children who suffer from early gastric ulcers with outlet obstruction shall raise the suspicion of the antral web. Complete obstruction madates early intervention. Around half of the cases with adequate feeding and growth need no intervention. Recurrent obstructive symptoms or adjacent ulcers justify a switch from observation to intervention to avoid complications or growth faltering.

## Introduction

Antral web is considered a rare cause of gastric outlet obstruction in children (1). The incidence is unclear, yet ever estimated to be 1–3 in 100,000 live births in the literature (2, 3). The pathogenesis includes *in-utero* vascular accident, failure of recanalization, or an excess of localized endodermal proliferation (1). This structural anomaly causes a spectrum of clinical presentations, depending on the degree of obstruction. In addition to the reported symptoms of persisted emesis, gastrointestinal bleeding, malnutrition, and failure to thrive, some may thrive with minimal signs and require no intervention at all. Surgical treatment options include simple dilatation, incision or excision of the web, antrectomy, or pylorectomy, *via* laparotomy or laparoscopy (3–6). The endoscopic intervention has attracted clinicians' interest due to minimal invasiveness, although the feasibility is not universal (3, 4).

With this wide variation of severity and disease course, pediatric patients' management guidance with the antral web is still lacking. Amid the kids under observational care, some encountered recurrent emesis, refractory peptic ulcer disease, or even life-threatening electrolyte imbalance and ultimately required surgery. The proper timing to intervene is a challenge for clinicians.

Herein, this study aimed to evaluate the presentations, diagnostic processes, treatment options, and outcomes of children diagnosed with antral web, based on a 20-year experience in a referral medical center. In addition, the clinical parameters of patients under a delayed intervention were further analyzed.

## Materials and Methods

### Patients

**Figure 1:**
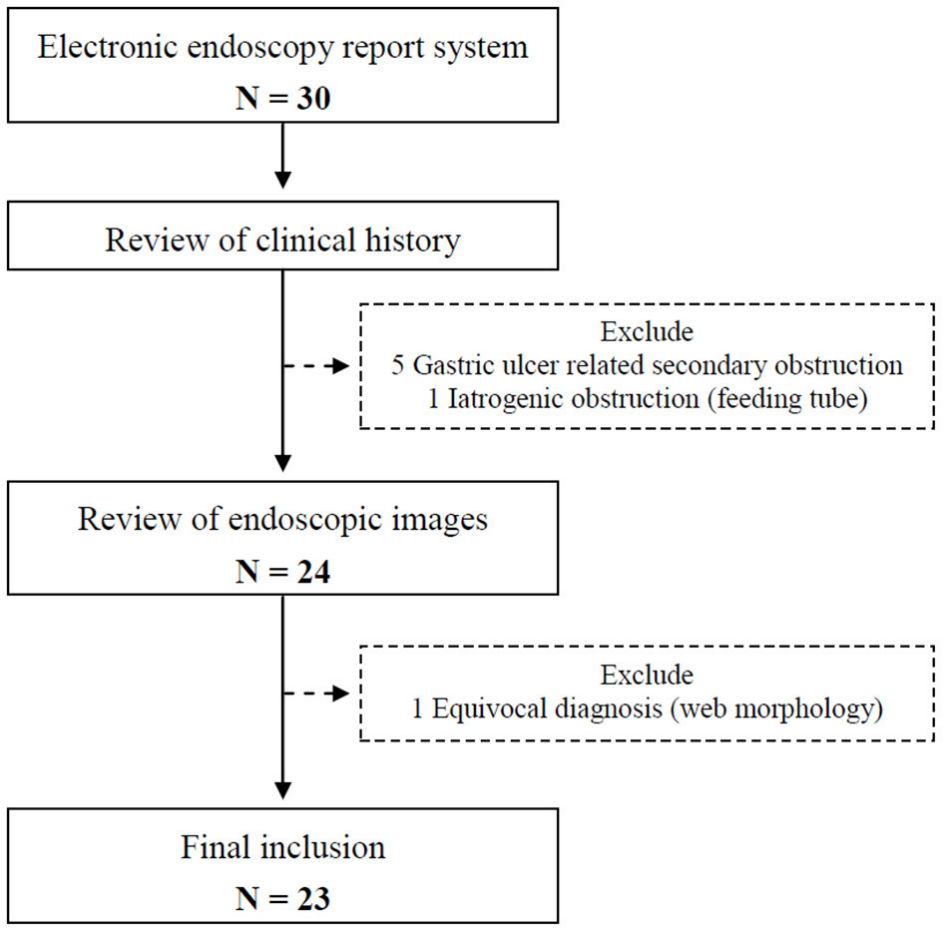
Flowchart showing the process of patient inclusion and exclusion.

This retrospective study comprised patients under 18 diagnosed with an antral web between Jan 2000 and Dec 2020 at the Department of Pediatrics, Chang Gung Memorial Hospital, Linkou branch, a referral medical center in Northern Taiwan. The electronic record of the endoscopic reports was searched utilizing keywords including “antral web,” “prepyloric web,” and “gastric web” to find out potential patients with the diagnosis. In addition, these likely patients were reviewed on their clinical history and endoscopic images to exclude false or ambiguous diagnoses (Figure 1). The study was approved by the Institutional Review Board of Chang Gung Memorial Hospital (IRB number: 202001986B0).

The antral web diagnosis was established with direct visualization of a web-like structure located at the antral or prepyloric area, with or without the accessory findings in abdominal sonography and upper gastrointestinal (UGI) series, which support the presence of gastric outlet obstruction. Patients with mimicking diagnoses were excluded, including pyloric spasm and infantile hypertrophic pyloric stenosis. Besides, those patients who had prior peptic ulcer disease and whose obstruction subsided after a course of acid-suppression therapy were considered secondary gastric outlet obstruction and were excluded. The index diagnostic esophagogastroduodenoscopy (IDE) indicates the first endoscopy identifying the finding of a web-like anomaly. The duration from diagnosis to intervention (DtI) is defined as the duration between the IDE and intervention (surgical and/or endoscopic treatment). Growth failure is defined as inadequate physical growth (<3^rd^ percentile) for age or growth deceleration across two major percentile lines on a World Health Organization (WHO) growth chart.

The medical charts and images of eligible cases were reviewed to collect demographics, clinical manifestations, diagnostic processes, medical treatment, intervention (surgical or endoscopic treatment), and outcomes. Patients with DtI longer than 30 days were further selected to assess their characteristics and courses.

### Literature Review

A literature review was performed to compare the treatment strategies. A search in the Pubmed database was conducted from January 1970 till January 2020 using the keywords “antral web” or “prepyloric web” and “children” or “pediatric.” Case series with English full text was retrieved. Demographics, intervals from onset to diagnosis and from DtI, and the proportion receiving the interventions (laparoscopy, laparotomy, and endoscopy) were the major parameters for comparison. Case reports and articles without the parameters mentioned above were excluded.

### Statistical Analysis

Data entry and processing were conducted using the Statistical Package for the Social Sciences (SPSS) v. 20 software (SPSS Inc., Armonk, NY, USA). Continuous data in normal distribution were expressed as mean ± standard deviation (SD); otherwise, they were described as the median and interquartile range (IQR). Categorical results were shown as frequencies.

## Results

### Demographic Data

A total of 23 cases were included after excluding five gastric ulcer-related secondary obstruction, one iatrogenic obstruction due to feeding tube placement, and one equivocal endoscopic morphology (Figure 1).

The male to female ratio was 1.3 to 1, and the median age of diagnosis was 10 months (Table 1). The majority had no specific past medical history, except for one with esophageal atresia (EA) and the other with combined immunodeficiency multiple intestinal atresias (CID-MIA) due to TTC7A (tetratricopeptide repeat domain 7A) mutation.

**Table 1:** Patient characteristics, initial presentations, and diagnostic tools.

**Characteristics**		
Gender (M:F)		1.3:1
Age (month)	Median (IQR)	10 (0.8–23)
	Range	0.2–116
**Initial presentation**		***N* (%)**
Vomiting		19 (83)
	Duration (month) (Median, IQR)	0.5 (0.2–1)
UGI bleeding		11 (48)
Abdominal pain		4 (17)
Shock		1 (4)
**Diagnostic tools**		***N* (%)**
Sonogram		20 (87)
	Gastric stasis	11 (55)
	Detection of web structure	3 (15)
UGI series		19 (83)
	Gastric outlet obstruction	18 (95)
	Detection of web structure	12 (63)
EGD		22 (96)
	Gastric ulcer	15 (68)
	Detection of web structure	22 (100)

*F, female; IQR, interquartile range; M, male; N, number; SD, standard deviation; UGI, upper gastrointestinal; EGD, esophagogastroduodenoscopy*.

### Clinical Manifestation

Patients commonly presented with vomiting (83%) and UGI bleeding (48%). The median duration of vomiting before the first admission was 0.5 months, with an IQR of 0.2–1 month. Antral web-related gastric ulcer hemorrhage manifested as hematemesis or melena, with some resulting in profound anemia. Abdominal pain appeared non-specific, possibly associated with ulcer or gastric distention. One patient developed hypotension as the initial presentation (Table 1).

### Diagnostic Workups

Anemia, electrolyte imbalance (hyponatremia, hypokalemia, hypochloremia), and metabolic alkalosis were corresponding laboratory abnormalities but non-specific to the diagnosis.

**Figure 2:**
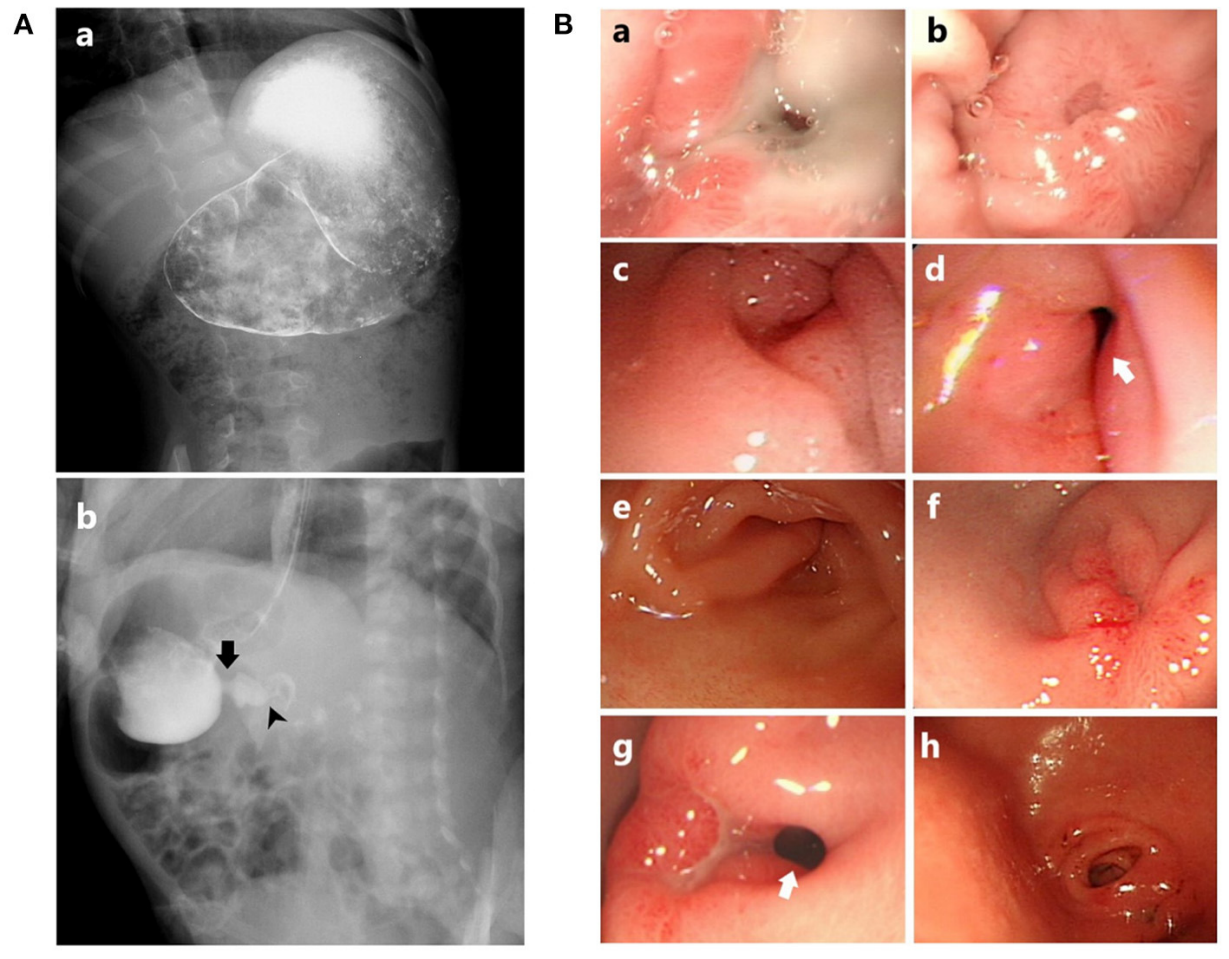
**(A)** Appearance of the antral web in UGI series: (a) Distended stomach, while the web structure is not demonstrated. However, the web is disclosed by endoscopy (the same patient as in **B**a,b); (b) A radiolucent septum-like projection, indicating the antral web (the same patient as in **B**c,d). Arrow, antral web; arrowhead, true pylorus. **(B)** Endoscopic appearance of the antral web: (a) Membrane-like web with ulcer involving a large portion of the web; (b) Healing status from (a), resulting in a complete obstruction; (c) Contracting web, forming a “pseudopylorus”; (d) Relaxation of the web and prepyloric area from (c), revealing the “true” pylorus; (e) A half-covered pylorus partially obstructed by a fan-like web; (f) A complete obstruction with an erosive antral web; (g) An antral web with a partially visible pylorus and an adjacent ulcer; (h) Antral web with a more complex zigzag of tunnel obscuring the pylorus. Arrow, true pylorus.

Over 80% of patients had received abdominal sonography or UGI series (Figure 2A). The positive rate of web detection by the UGI series was 63%, while only 15% by the sonogram. Features of gastric stasis or outlet obstruction were described in about half of the cases with these two modalities.

Except for one case who was diagnosed with the UGI series and received postoperative endoscopic follow-up, all the other patients have received EGD to confirm the presence of the antral web (Figure 2B). Concurrent gastric ulcers were commonly found [68% (15/22)], with the majority located at the antrum and pre-pyloric area, partially involving the web. No *Helicobacter pylori* (Hp) infection was documented. The median duration between the admission date to the performance of IDE was 3 days. All the IDE were the first EGD during the diagnostic process, indicating that none required a second EGD to yield the impression.

### Medical Treatment

Ten patients (43.5%) received only medical treatment during the whole follow-up period. Acid suppressive therapy, either histamine antagonist or proton pump inhibitor (PPI), was applied to around 70% of cases, which correlated to the proportion of concurrent peptic ulcer (Table 2). Parenteral nutrition was given in nine patients, including four younger than a month old; five were managed with interventions at last while the others resumed enteral nutrition. None of our patients was treated with antispasmodics, curd formula, or permanent enteric tube feeding. Six patients who received medical treatment alone were faltered [body weight (<3rd percentile)] at the time of diagnosis, yet all of them caught up with acceptable anthropometric parameters at the last follow-up.

**Table 2:** Medical and interventional (surgical, endoscopic) treatments.

**Medical treatment**		***N* (%)**
H2RA		12 (52)
Proton pump inhibitor		15 (65)
Total parenteral nutrition		9 (39)
**Intervention**		***N*** **(%)**
Antrectomy	Laparoscopy	3 (13)
	Laparotomy	2 (9)
Web excision/incision	Laparoscopy	4 (17)
	Laparotomy	4 (17)
Endoscopic procedure	Electrocauterization	2 (9)
	Balloon dilatation	2 (9)

*H2RA, histamine 2 receptor antagonist; N, number*.

### Intervention (Surgical and/or Endoscopic Treatment)

A total of 13 patients underwent interventions, including surgeries in 12 patients and endoscopic treatment in two (both interventions in one) (Table 2). The median DtI was 10 days (IQR 7.25–219 days). There was no use of local corticosteroid or botulinum toxin injection. In the intervention group, 23.1 and 15.4% had low body weight (<3^rd^ percentile) and low body height (<3^rd^ percentile) at the time of diagnosis, respectively. These anthropometric parameters were not statistically different between the intervention and exclusive-medical treatment groups.

Endoscopic treatment was performed in two cases. The first case was initially treated with two surgeries at another hospital (web excision at 5 days old and pyloroplasty at 6 months old), with recurrent stenosis 1 month later. His symptoms resolved after a session of endoscopic electrocauterization with balloon dilatation (7). The second case was a 2-year-4-month-old boy who received a total of two and seven sessions of endoscopic electrocauterization and balloon dilatations, respectively. However, he was lost to follow-up when being referred to surgery for the persistent antral web with recurrent ulcers.

Surgical procedures varied, including eight web excision/incision (four laparotomy and four laparoscopy) and five antrectomies (two laparotomy and three laparoscopy). One patient required an antrectomy as a second operation 3 months after prior web resection, and another underwent endoscopic management for postoperative restenosis, as depicted above. No surgery-associated complication was encountered, such as dumping syndrome. The average interval between operation and re-feeding was 3 days.

### Patients With Prolonged DtI

Among the 13 cases with interventions, five had relatively prolonged DtI, up to 755 days (Table 3). Among these five patients, the mean age at diagnosis was 18.5 months, without gender preponderance. The initial presentations and endoscopic findings were not distinguishable from the others.

**Table 3:** Cases with prolonged interval from diagnosis to intervention.

	**Sex**	**Age** **(mo)**	**Initial presentation**	**IDE**	**Causes of postponement**	**Causes of intervention**	**DtI**	**Intervention**	**Outcome**
1	F	22	Vomiting for 10 days	Suspect antral web: Obstruction of pylorus Gastric ulcer (prepyloric)	Acceptable intake. No severe vomiting Failure to thrive (–) UGI series: Patent without cGOO	Recurrent vomiting	754	Laparoscopic web excision	R (+)
2	M	17	Vomiting for 4 days Tarry stool	Suspect antral web: Obstruction of pylorus Gastric ulcer (prepyloric)	Acceptable intake. No severe vomiting Failure to thrive (–)	Persisted web Recurrent ulcer	479	Laparoscopic web excision	R (+)
3	M	28	Abdominal pain Tarry stool	Suspect antral web: Gastric ulcer (antral)	Acceptable intake. No severe vomiting Failure to thrive (+) UGI series: Refused by family	Persisted pain Failure to thrive Poor response to PPI	282	Endoscopic electrocauterization and balloon dilatation	R (–)
4	M	8.7	Vomiting for 8 months	Suspect antral web: Partial obstruction of pylorus	Acceptable intake. No severe vomiting Failure to thrive (–) Esophageal atresia	Combined operation with fundoplication	650	Laparoscopic web excision	R (+)
5	F	17	Vomiting for 4 days Coffee-ground vomitus Tarry stool	Suspect antral web: Partial obstruction of pylorus Gastric ulcer (web)	Acceptable intake. No severe vomiting Failure to thrive (–)	Recurrent vomiting Electrolyte imbalance Severe dehydration	30	Laparoscopic antrectomy	R (+)

*cGOO, complete gastric outlet obstruction; DtI, diagnosis to intervention; F, female; IDE, index diagnostic esophagogastroduodenoscopy; M, male; mo, month; PPI, proton pump inhibitor; R, resolution of symptoms; UGI, upper gastrointestinal*.

Except for one case (EA post-repair with feeding intolerance at neonate, gastric outlet obstruction diagnosed by UGI without immediate operation; fundoplication for reflux esophagitis and antrectomy for the antral web at 2.5 years of age), the rest four patients were postponed owing to tolerable gastric outlet obstruction. These patients underwent multiple EGDs that disclosed recurrent gastric ulcers without worsening outlet obliteration. One suffered from failure to thrive before the intervention. The feeding status appeared acceptable without evident emesis or bleeding. In retrospect, these “postponements” reflected family's preferences and the trade-offs between surgical invasiveness and compensatory feeding tolerance.

The recurrence of obstructive symptoms or ulcers was the main reason for switching to intervention. Unfortunately, one patient developed near-total occlusion after ulcer healing, leading to severe dehydration and electrolyte imbalance.

### Outcomes

Overall, no disease-associated mortality was confronted. The median duration of follow-up was 1,135 days (IQR 148.5–1817 days). Clinical remission was not documented in two cases: one was transferred to a second hospital due to personal preference, and the other (the second case treated by endoscopy) lost to follow-up after the referral to the surgical department.

Among patients with concurrent gastric ulcers (*N* = 15), five achieved symptom resolution with medical treatment while the rest eventually required interventions, six immediately but four delayed (nine surgical and one endoscopic treatment) due to recurrent or refractory disease.

### Literature Review

Six pediatric series in the English literature containing 78 cases were retrieved and compared (1, 3, 5, 6, 8, 9) (Table 4). In addition to the gender and diagnostic age, the onset-to-diagnosis and modalities of intervention were denoted. However, the DtI was merely addressed in one of these studies.

**Table 4:** Comparisons of pediatric case series of antral web.

	**Year**	**No**.	**Country**	**M:F**	**Diagnostic age**	**Max. OtD**	**DtI**	**Intervention**	**Reference**
1	1978	28	USA	1.54:1	86% <6 m/o, Max. 8.3 y/o	14 wk	NA	85.7% (24/28) Laparotomy operation	(1)
2	1980	11	USA	1.2:1	Mean: 3.7 m/o Range: 1–8.5 m/o	8 mo	NA	63.6% (7/11) Laparotomy operation	(8)
3	1987	6	USA	NA	Range: 4 d/o–11 y/o	1 yr	Range: 4 mo−6 yr	100% (6/6) Operation (6)	(9)
4	2013	3	China	3:0	Mean: 9 m/o Range: 31 d/o−17 m/o	16 mo	NA	100% (3/3) Laparoscopic operation	(5)
5	2019	21	USA	2:1	Mean: 30 m/o Range: 0.7–142 m/o	11 yr	NA	100% (21/21) Laparotomy (19)/Laparoscopy (2)	(6)
6	2019	9	Austria	3.5:1	Median: 4 y/o Range: 1 d/o−11 y/o	11 yr	NA	100% (9/9) Operation (6) /Endoscopy (3)	(3)
7	2020	23	Taiwan	1.3:1	Median: 10 m/o Range: 0.2–116 m/o	8 mo	Median: 10 d IQR: 7.25–219 d	56.5% (13/23) Operation (12) /Endoscopy (2)	Present study

*d, day; d/o, day-old; DtI, diagnosis to intervention; IQR, interquartile range; M:F, male to female ratio; mo, month; m/o, month-old; Max, maximum; NA, not available; No, case number; OtD, onset to diagnosis; wk, week; yr, year; y/o, year-old*.

## Discussion

This study demonstrated the two-decade experience of managing pediatric antral web from a single referral center in Taiwan. The present report is the most extensive case series of Asian children in the literature. The results revealed the diverse natural courses of the disease and reflected a dilemma in conservative medical treatment vs. interventions.

The antral web can originate from a congenital structural anomaly or the secondary result of local inflammation and fibrosis, such as peptic ulcer disease. These two categories are not mutually exclusive. The partially obstructive congenital antral web could be overlooked and misdiagnosed as peptic stricture, and superimposed ulcers could aggravate the obstruction (10). We excluded patients with gastric outlet obstruction secondary to chronic peptic ulcers. Presumably, our case series presented a precious experience in managing the early or so-called “congenital” antral web.

In demographic characteristics, a male predominance was observed (Tables 1, 4). The oldest was 142-month-old at diagnosis, while the majority was around infancy. A review-based series covering 65 patients reported before 2,000 stated that around 70% of cases were newborns or infants at diagnosis (11).

The presenting symptoms included emesis, abdominal fullness, abdominal pain, feeding intolerance, failure to thrive, and unexplained irritable crying (11). Emesis was the most prevalent presentation for cases under 6 months old, while the manifestations for patients beyond infancy appeared diverse, intermittent, and non-specific. In addition to the common symptoms, our study observed a higher proportion of UGI bleeding (48%), in contrast to no gastric ulcers in some series (1, 3, 5, 6). Given the rarity of gastrin secreting tumors and Hp infection in this age, obstruction-related acid stasis is hypothesized as the cause of ulceration. Pediatric peptic ulcers primarily develop in the second decade, and a study focusing on Taiwanese children disclosed a median age of diagnosis for peptic ulcer disease at 11.4 years (12). Besides, commonly associated etiologies are Hp infection and non-steroid anti-inflammatory drugs (NSAIDs) usage (13). Antral web-related gastric ulcer is not regarded as a leading cause. Nevertheless, in a young child presenting gastric ulcers with outlet obstruction, recurrent or refractory to medical therapy, it shall alert the clinician of the congenital antral web.

The diagnosis can be challenging, given the non-specific symptoms and relatively insufficient awareness of this etiology (3). The inconsistency of episodic presentations and misinterpretation of the image may also puzzle the physicians (3). Partial obstruction may lead to a prolonged diagnostic process and inefficacious treatment (5). The interval between the onset and the definite diagnosis could take as long as a decade (3, 6).

Sonography and fluoroscopy studies are two complementary tools for endoscopy, which help differentiate mimicking diagnoses of gastric outlet obstruction. However, the sensitivity of web detection in both examinations seems unsatisfactory in our series. The suboptimal contrast filling, skills of the operator, and the cooperation of patients were attributed limitations (6). In our series, EGDs detected web structure accurately and evaluated the occurrence of ulcers, although some prior reports did not find EGD as valuable as expected (5, 14).

Around 43.5% of patients sustained tolerable conditions without intervention, a relatively higher proportion than other series (28.6–36.4%) (1, 8). Feeding tolerance was acceptable, and the follow-up EGDs also confirmed the luminal patency in these non-intervention patients, further supporting the observational management. For those who underwent intervention, the final confirmed outcomes were generally good. The elimination of web tissue in web excision/ incision was often inadequate due to the restricted operative field, difficulty of margin determination, and variable technique; hence, antrectomy was more favored in our later practice. Furthermore, the laparoscopic method has been increasingly applied recently. Although residual web tissue was observed in a few patients, they remained symptom-free. As of endoscopic treatment, several studies have demonstrated both the strength and limitations (2–4, 10). Endoscopic treatment requires suitable instrumentation, technical expertise, and multidisciplinary support (3). Besides, multiple sessions are necessary to achieve sustainable symptom control (6). In this series, endoscopic therapy was performed in only two cases. It takes more experience to polish the technique and clarify the benefit over surgical intervention.

The intervention timing is critical but rarely discussed. In clinical scenarios, physicians may face a dilemma between watchful waiting and immediate intervention in patients with partial obstruction or complicated ulcers. Bell et al. proposed that patients with mild to moderate symptoms or non-obstructing web could take conservative measures since the resolution of symptoms is still anticipatable (1). The DtI ranged from months to years among our five cases who finally received interventions. Although growth failure was only noted in one case, they all manifested with unremitted symptoms. Moreover, patient 5 relapsed with a rapid deterioration within weeks. Only one retrieved case series in literature elaborated this issue of “delay in treatment,” reporting a range of DtI up to 6 years. Among their four cases with prolonged DtI, major concerns included inadequate initial workup, initially incorrect diagnosis, and expectant conservative management (9). The symptom severity presumably could be associated with the web's morphology, the impact of adjacent ulcers, and the individual's compensatory capacity. Due to good postoperative outcomes, delayed intervention may seem meaningless and risk-taking for hypovolemia, electrolyte imbalance, failure to thrive, or recurrent ulcer bleeding.

Even though this is the largest and latest series for Asian children with congenital antral web, this study still has several limitations due to its retrospective and single-center nature. The performance and safety issues of variable treatment modalities require further comparison. More longitudinal observations and a larger cohort will be needed to answer the “optimal” management.

## Conclusion

Antral web is a rare etiology of gastrointestinal obstruction/peptic ulcer bleeding in children, with a spectrum of manifestation related to the level of luminal narrowing or combined ulceration. Children suffering from the early occurrence of ulcers with gastric outlet obstruction shall raise the suspicion of this diagnosis instead of primary peptic ulcer disease. Around half of the cases need no intervention due to a tolerable partially obstructive web. Surgical treatments generally accomplish satisfactory outcomes, while the effect of endoscopic intervention mandates more experience. For those patients adopting conservative management, recurrent obstructive symptoms may justify the timing for intervention.

## Data Availability Statement

The original contributions presented in the study are included in the article/supplementary material, further inquiries can be directed to the corresponding authors.

## Ethics Statement

The studies involving human participants were reviewed and approved by Institutional Review Board of Chang Gung Memorial Hospital (IRB number: 202001986B0). Written informed consent from the participants' legal guardian/next of kin was not required to participate in this study in accordance with the national legislation and the institutional requirements.

## Author Contributions

P-JY, J-YL, and M-WL: study conception, design, analysis, and data interpretation. P-JY, H-CC, C-CC, and M-WL: data acquisition. P-JY: drafting of the manuscript. M-WL: critical revision. All authors approved the final approval.

## Conflict of Interest

The authors declare that the research was conducted in the absence of any commercial or financial relationships that could be construed as a potential conflict of interest.

## Publisher's Note

All claims expressed in this article are solely those of the authors and do not necessarily represent those of their affiliated organizations, or those of the publisher, the editors and the reviewers. Any product that may be evaluated in this article, or claim that may be made by its manufacturer, is not guaranteed or endorsed by the publisher.
